# Direct inhibition of the TLR4/MyD88 pathway by geniposide suppresses HIF‐1α‐independent VEGF expression and angiogenesis in hepatocellular carcinoma

**DOI:** 10.1111/bph.15046

**Published:** 2020-04-12

**Authors:** Cheng Zhang, Ning Wang, Hor‐Yue Tan, Wei Guo, Feiyu Chen, Zhangfeng Zhong, Kwan Man, Sai Wah Tsao, Lixing Lao, Yibin Feng

**Affiliations:** ^1^ School of Chinese Medicine, Li Ka Shing Faculty of Medicine The University of Hong Kong Hong Kong S.A.R. China; ^2^ Department of Surgery, Li Ka Shing Faculty of Medicine The University of Hong Kong Hong Kong S.A.R. China; ^3^ School of Biomedical Science, Li Ka Shing Faculty of Medicine The University of Hong Kong Hong Kong S.A.R. China

## Abstract

**Background and Purpose:**

As a typical hypervascular tumour, hepatocellular carcinoma (HCC) is predominantly grown through angiogenesis. Geniposide is a promising anti‐inflammatory compound found in *Gardenia jasminoides*, but its effects on the progression of HCC remain untested.

**Experimental Approach:**

The anti‐HCC effects of geniposide was investigated in cellular models and orthotopic HCC mice. Transcriptional regulation of the VEGF promoter was measured by dual‐luciferase reporter assay. The anti‐angiogenic action of geniposide was measured by tube formation assay. Both surface plasmon resonance techniques and human phospho‐kinase array analysis were utilized to validate the relationship between targets of geniposide and hepatocarcinogenesis.

**Key Results:**

Geniposide exhibited significant disruption of HCC proliferation, invasion, angiogenesis and lung metastasis in orthotopic HCC mice. Geniposide inhibited secretion of VEGF by HCC and suppressed the migration of endothelial cells and the formation of intra‐tumour blood vessels, without cytotoxicity and independently of the transcription factor HIF‐1α. Direct inhibition of TLR4 by geniposide led to the shutdown of the TLR4/MyD88 pathway and STAT3/Sp1‐dependent VEGF production. However, LPS, an agonist of TLR4, restored STAT3/Sp1‐related VEGF production in geniposide‐inhibited HCC angiogenesis.

**Conclusion and Implications:**

The direct inhibitory effect of geniposide on TLR4/MyD88 activation contributes to the suppression of STAT3/Sp1‐dependent VEGF overexpression in HCC angiogenesis and pulmonary metastasis. This action of geniposide was not affected by stabilization of HIF‐1α. Our study offers a novel anti‐VEGF mechanism for the inhibition of HCC.

**Abbreviations**
HCC: hepatocellular carcinoma
MyD88: myeloid differentiation primary response 88
Sp1: specificity protein 1
TLR4: toll‐like receptor 4

**What is already known**

- Progression of hepatocellular carcinoma is known to involve VEGF‐induced angiogenesis which is dependent on HIF‐1α.
- Geniposide exerts many anti‐inflammatory effects, but its effects on angiogenesis in hepatocellular carcinoma are untested.

**What this study adds**

- A novel anti‐VEGF mechanism, involving the TLR4/MyD88 pathway, to inhibit progression of hepatocellular carcinoma .
- Geniposide suppresses VEGF expression and angiogenesis in hepatocellular carcinoma, independently of HIF‐1α.

**What is the clinical significance**

- Novel therapeutic targets for VEGF inhibition in hepatocellular carcinoma may improve pharmacological management of cancer.
- Geniposide, a potential and affordable anti‐VEGF compound, shows promise as clinical treatment for hepatocellular carcinoma.

## 1 INTRODUCTION

Hepatocellular carcinoma (HCC), a common and lethal form of cancer, results in an annual mortality of one million people worldwide. The high death rate reflects the unpredictable manifestations of HCC in the early stage and poor prognosis in the advanced stages of this condition (Sheng, Qin, Zhang, Li, & Zhang, 2018). Notably, only 30% of HCC patients are suitable for surgical therapy, including liver transplantation (Santambrogio et al., 2016). As a typical hypervascular cancer, HCC relies on angiogenesis to grow and metastasize. Accordingly, treatment of HCC has adopted an anti‐angiogenic approach, by blocking the expression of the VEGF family of ligands and receptors (Liu et al., 2018). The multi‐kinase inhibitor sorafenib has been approved as the first‐line therapy for advanced‐stage HCC with its anti‐angiogenic effect. However, its benefits are transient due to the worsening hepatic dysfunction and promoting regrowth (Fang et al., 2018). Thus, there is a pressing need for a novel therapeutic mechanism and new compounds for the suppression of HCC growth and metastasis.

In HCC, the production of VEGF facilitates the recruitment of endotheliocytes and enhances vascular permeability. Subsequently, angiogenesis generates an increased supply of nutrition and of metastatic HCC cells (Chesnokov et al., 2018). VEGF is a principal factor for abnormalities in vascular structure and function, contributing to HCC angiogenesis and metastasis (Zhang, Zhang, et al., 2018). Aberrant expression of VEGF can be directly involved in the pathological aspects of HCC (Deng et al., 2013). The level of VEGF is proportional to the progression of HCC, including tumour invasion, metastasis and differentiation (Jin et al., 2017). This pathological mechanism provides a robust theoretical basis for the anti‐angiogenic strategy of controlling HCC progression, by VEGF inhibition.

Geniposide (PubChem CID: 107848) is an iridoid glycoside, abundantly available in the herb *Gardenia jasminoides*, and is notable for treating various diseases with inflammation or oxidative stress, including brain disorders, atherosclerosis and diabetes (Cheng et al., 2019; F. Li et al., 2016; Pan et al., 2019; Shan et al., 2017). However, scientific investigations focusing on its anti‐tumour property are scanty, especially in HCC. Regarding its reported anti‐HCC studies in nearly two decades, geniposide suppresses HepG2 and Huh7 cells by regulating miR‐224 via blocking the Wnt/β‐catenin and Akt cascades (Yu, Wang, Tao, & Sun, 2019). Also, aflatoxin B_1_‐induced HCC in the rat was improved by geniposide through inhibiting γ‐glutamyl transpeptidase activity (Lin et al., 2000). Our previous studies have demonstrated the suppressive effect of its aglycone, genipin, on HCC through inhibiting intrahepatic metastasis and regulating the tumour‐associated macrophages (Tan et al., 2016; N. Wang et al., 2012). Notably, an earlier study showed that geniposide exhibited an anti‐angiogenic effect in the chick embryo chorioallantoic membrane assay (Koo et al., 2004). However, whether geniposide inhibits HCC through its anti‐angiogenic action remains unknown.

In the present study, we evaluated the inhibitory effect of geniposide on the development of HCC, in vivo and in vitro, including the proliferation, invasion, metastasis and angiogenesis of HCC.

## 2 METHODS

### 2.1 Cell lines and cell cultures

Human HCC cell line PLC/PRF/5 cells and HUVECs were purchased from ATCC (USA). The luciferase reporter‐tagged MHCC‐97L cells were obtained as a gift by Prof. Man Kwan from the Department of Surgery, The University of Hong Kong. HUVECs were cultured in EGM‐2 complete medium (Lonza, Switzerland). Both PLC/PRF/5 and MHCC‐97L were maintained in DMEM with 4.5% glucose, 10% FBS and 1% penicillin/streptomycin (Gibco, USA) in humidified condition containing 5% CO_2_ at 37°C. The normoxic condition refers to the standard condition (20% oxygen), whereas cells incubated in a nitrogen/carbon dioxide/oxygen chamber with 1% oxygen represented the hypoxic condition.

### 2.2 Orthotopic HCC implantation in athymic nude mouse

All animal care and experimental protocols were approved by the Committee on the Use of Live Animals in Teaching and Research, The University of Hong Kong (Ref: 4251‐17). Animal studies are reported in compliance with the ARRIVE guidelines (Kilkenny, Browne, Cuthill, Emerson, & Altman, 2010) and with the recommendations made by the *British Journal of Pharmacology*.

A model of orthotopic HCC‐implanted mice was established as described in our previous report (Wang, Han, et al., 2017). In brief, a solid HCC tumour was formed within 2 weeks by a subcutaneous injection of 5 × 10^6^ luciferase‐labelled MHCC‐97L cells into the lateral abdomen of nude mice. Afterwards, cluster‐shaped HCCs were cut into 1‐mm^3^ pieces followed by transplantion into the left lobe of a nude mouse liver. Seven days after implantation, mice were given geniposide (Neautus, China; 30 mg·kg^−1^ for 2 days, intragastrically) and/or a single i.p. injection of LPS (3 mg·kg^−1^; Sigma‐Aldrich, USA) at the beginning of a 5‐week treatment. LPS was utilized as an agonist of toll‐like receptor 4 (TLR4; Pizzuto et al., 2019). Growth of the HCC was monitored, once a week, by luciferase imaging analysis, using the IVIS Spectrum system (PerkinElmer, USA). All the mice were killed via an overdose injection of pentobarbitone (200 mg·kg^−1^) at the end of the experiment.

### 2.3 Histology and immunofluorescence

For the histological study, paraffin‐embedded sections (5 μm) were collected on the surface of the slides followed by de‐waxing in xylene and rehydrated by alcohol with gradient concentration from 100% to 70% (10% per interval). After the processing of rehydration, slides were incubated with haematoxylin and 0.25% eosin for 5 and 1 min, respectively (Sigma‐Aldrich, USA). Afterwards, slides were immersed in Canada balsam (Sigma‐Aldrich, USA) followed by the observation of haematoxylin and eosin‐stained HCC morphology with a BX43 light microscope (Olympus, Japan; *n* = 5 per group).

The immuno‐related procedures used comply with the recommendations made by the *British Journal of Pharmacology.* For the immunofluorescent analysis, staining in tissue is slightly different from that in the cell. Both de‐waxing and antigen retrieval steps were applied in the tissue staining alone. Other staining steps are identical. For details, the de‐waxed sections (*n* = 5 per group) were immersed in 10‐mM citrate buffer (Sigma‐Aldrich, USA) for antigen retrieval, followed by incubating with 10% goat serum for 30 min for blocking procedure. Then, sections were incubated for 12 h at 4°C with anti‐glypican‐3 (Cat: ab66596, Abcam, USA), anti‐HIF‐1α (Cat: 36169, CST, USA), anti‐Ki67 (Cat: 2586, CST, USA), anti‐CD31 (Cat: 3528, CST, USA), anti‐MyD88 (Cat: 50010, CST, USA), anti‐Sp1 (Cat: 9389, CST, USA) and anti‐STAT3 (Cat: 9139, CST, USA), respectively (Cell Signaling Technology, USA). Afterwards, slides were counterstained with corresponding secondary antibody Alexa Fluor 488 (Cat: A32723, Invitrogen, USA), Aleca Fluor 568 (Cat: A‐11031, Invitrogen, USA) and DAPI (Cat: D1306, Invitrogen, USA), followed by mounting with fluorescent mounting medium (Dako, Denmark). All the specimens were visualized by using the confocal microscope (Carl Zeiss LSM 780, Germany).

### 2.4 Bioinformatics study

Based on the analysis of human phospho‐kinase array, the underlying mechanism of geniposide‐induced HCC inhibition was predicted by bioinformatics analysis, as in our previous studies (Wang, Tan, Li, & Feng, 2017; Zhang, Wang, et al., 2018). In brief, differentially expressed genes were imported into Gene Ontology (GO) and Kyoto Encyclopedia of Genes and Genomes (KEGG) analysis followed by the identification of the contributive targets and gene‐rich terms according to the enrichment analysis on the platform of DAVID (available at https://david.ncifcrf.gov). In particular, GO analysis for target mining is composed of three ontologies, including biological process, molecular functions and cellular components. GlueGo‐based network pharmacology was constructed by Cytoscape (access at http://www.cytoscape.org/). To enhance the functionality of target prediction, cluster tree analysis was applied by SPSS Statistics (SPSS Inc., USA) and RStudio (R Inc., USA). The correlation degree of –log_10_ (*P* value) from each identified geniposide‐regulated pathway was imported into SPSS for transforming all variables to *Z* scores by choosing the method of squared Euclidean distance, which aimed to yield equal metrics and weighting value. Next, the hierarchical cluster membership was defined by SPSS‐dependent weighted cluster analysis, followed by further identifying homogeneous biological terms in each cluster. Finally, the R programming visualization was performed by RStudio to circle‐hierarchically show the outcomes.

### 2.5 Plasmid and transfection

The plasmids created by research colleagues were available on the platform of Addgene, including pGL4.10‐VEGFprom (pGL4.10 expressing luciferase‐based reporter in VEGF promoter region), pN3‐Sp1FL (pN3 vector for hyperactivation of SP1) and EF.STAT3C.Ubc.GFP (lentiviral expression of constitutively active STAT3). In detail, the contributors of these specific plasmids were listed as follows: the pGL4.10‐VEGFprom was a gift from David Mu (Addgene Plasmid # 66128; http://n2t.net/addgene:66128; RRID:Addgene_66128; Wood et al., 2016); the pN3‐Sp1FL was a gift from Guntram Suske (Addgene Plasmid # 24543; http://n2t.net/addgene:24543; RRID:Addgene_24543); and the EF.STAT3C.Ubc.GFP was a gift from Linzhao Cheng (Addgene Plasmid # 24983; http://n2t.net/addgene: 24983; RRID:Addgene_24983; Hillion et al., 2008). Transfection was performed according to the manufacturer's instruction regarding the application of FuGene reagent (Promega, USA). In brief, nucleotide was mixed with FuGene transfection reagent in the serum‐free DMEM medium. After 15‐min incubation at room temperature, cells were transferred to the mixture for another 48‐h incubation. Treatments would be applied following the above procedures.

### 2.6 Real‐time quantitative PCR

Total RNA from HCC cells was extracted and purified by using TRIzol (Takara, Japan; *n* = 5 per group). PrimeScript RT master mix (Takara, Japan) was used to reverse transcription. Quantitative PCR was conducted by using SYBR Green Probe (Takara, Japan) and designed corresponding primers on a Light Cycler 480 PCR System (Roche, Switzerland). The sequences of isoform‐specific primers were shown as follows: VEGF‐A forward: CCTTGCCTTGCTGCTCTAC, VEGF‐A reverse: TTCTGCCCTCCTCCTTCTG; TLR‐4 forward: TTGTATTCAAGGTCTGGCTGG, TLR‐4 reverse: GCAACCTTTGAAACTCAAGCC; and β‐actin forward: GCTTCTCCTTAATGTCACGC, β‐actin reverse: CCCACACTGTGC CCATCTAC. Calculation of the relative transcriptional expression of the target genes was performed after standardization with the internal reference β‐actin transcript.

### 2.7 Flow cytometry

The assays for programmed cell apoptosis and necrosis were performed according to the instructions of the Annexin V Apoptosis Detection Kit (BD Biosciences, USA). Briefly, the in vitro HCC cells, including MHCC‐97L and PLC/PRF/5, were treated under normoxic or hypoxic condition after 24‐h geniposide or vehicle treatment followed by washing in the binding buffer. Both HCC cells were respectively stained with PE Annexin V and 7‐AAD for 15 min. The percentage of alive, apoptotic and necrotic cells were determined by flow cytometer (BD Biosciences, USA).

### 2.8 Quantifying VEGF secretion by elisa assay

In accordance with the manufacturer's instruction, concentrations of HCC‐secreted VEGF were detected by VEGF enzyme‐conjugated immunosorbent assay kit (Excell Biology, China). Cell supernatant was transferred to a microplate that was coated with an anti‐VEGF antibody. Afterwards, HRP‐linked streptavidin was added in the plate and incubated for 30 min prior to the addition of TMB substrate solution. At the end of the experiment, the enzyme/substrate reaction was terminated by the stop buffer. VEGF concentration was determined at 450‐nm absorbance (*n* = 5 per group).

### 2.9 Immunoblotting

HCC cells were lysed by RIPA buffer (Thermo Fisher Scientific, USA). Protein concentrations were determined by Bradford protein assay. Equal amounts of proteins were separated on SDS‐PAGE (CA) followed by electro‐transferring on the PVDF membrane (Bio‐Rad, CA). After finishing the electro‐transfer, membranes were immersed in the blocking buffer containing 5% BSA for 2 h prior to the incubation with primary antibodies, including anti‐TLR4 (Cat: MAB6248, R&D Systems, USA), anti‐MD2 (Cat: ab24182, Abcam, USA), anti‐glypican‐3 (Cat: ab66596, Abcam, USA), anti‐Sp1 (Cat: 9389, CST, USA), anti‐STAT3 (Cat: 9139, CST, USA), anti‐phospho‐STAT3 (Cat: 9145, CST, USA), anti‐HIF‐1α (Cat: 36169, CST, USA), anti‐phospho‐p38 MAPK (Cat: 4511, CST, USA), anti‐IκB‐α (Cat: 4812, CST, USA), anti‐p65 (Cat: 8242, CST, USA) and anti‐MyD88 (Cat: 50010, CST, USA), respectively. HRP‐conjugated secondary antibodies, including anti‐rabbit IgG (7074, CST, USA) and anti‐mouse IgG (7076, CST, USA), were correspondingly used to the incubate the membranes for 2 h (Cell Signaling Technology, USA). Bands were visualized under the chemiluminescence system (Bio‐Rad, USA).

### 2.10 Co‐immunoprecipitation

Dynabeads® Co‐immunoprecipitation Kit (Invitrogen, USA) was used for measuring the coupling of protein complexes. Briefly, after cells had undergone various treatments, cells were lysed by NP‐40 cell lysis buffer (Life Technologies, USA). The target protein was precipitated with antibodies that are covalently coupled to Dynabeads, which was followed with the elution (PBS in 0.1% Tween 20). The specific protein–protein complex was pulled down and separated by SDS‐PAGE. The chemiluminescence system (Bio‐Rad, USA) was applied for the visualization and quantification of the target proteins.

### 2.11 Migration assay

A co‐culture system was established to assess the cross‐biological activity of HCC‐induced HUVEC migration. HUVECs were inoculated in the donor chamber, whereas PLC/PRF/5 cells were seeded in the receiving chamber. Both cells were maintained in the serum‐deprived medium condition for 6 h. After then, HUVECs and geniposide‐treated PLC/PRF/5 were cultured in the complete medium under either normoxic or hypoxic condition at 37°C for 24 h. Next, the remaining cells located in the donor insert were removed by a cotton ball, while cells at the receiving chamber were fixed by 4% paraformaldehyde (PFA) for 2 h followed by staining with crystal violet. The number of migrated HUVECs was quantified by ImageJ (NIH, USA) in five random fields of each well. Representative images were captured via a microscope (EVOS, USA).

### 2.12 Endothelial cell tube formation assay

Each well of a 24‐well plate was coated with 300‐μl basement membrane matrix with reduced growth factor (Invitrogen, USA) for 30 min at 37°C prior to seeding with HUVECs with complete (EGM‐2) or conditioned medium. PLC/PRF/5‐culture medium (DMEM for 24 h) with or without either LPS (100 ng·ml^−1^) or recombinant VEGF (re‐VEGF) treatment (20 ng·ml^−1^, 293‐VE, R&D Systems) were prepared as the conditional media according to the published paper (Bhattacharya et al., 2016). After setting the matrix, HUVECs (JCRB Cell Bank, Japan) were seeded in each well (approximately 4 × 10^4^ cells per well) and incubated in various conditional media for 6 h. Then, each well was washed with PBS followed by adding 4% PFA for 1 h at room temperature. After removing 4% PFA, the figures of tube formation were captured by a microscope (EVOS, USA). Quantification of tubular networks, including total branching length and number of tubes, was performed by ImageJ with Angiogenesis Analyzer plugin (NIH, USA) in six independent wells.

### 2.13 Dual‐luciferase assay

The determination of the activity of the VEGF promoter reporter was carried out according to the manufacturer's instruction (Promega, USA). In brief, PLC/PRF/5 cells were transfected with pGL4.1 containing the VEGF promoter region (−1,000 to −1) for 48 h. Afterwards, cells were lysed and then quantified the Firefly and Renilla luciferase values on a fluorescence plate reader (BMG LABTECH, Germany). Firefly luciferase signal was analysed by the normalization of the Renilla luciferase signal (*n* = 5 per group).

### 2.14 Proteome profiler human phospho‐kinase array

Total proteins (100 μg) were extracted from PLC/PRF/5 cells with or without 24‐h geniposide treatment in the normoxic condition. The lysed proteins were transferred to the human phospho‐kinase array (R&D Systems, USA). Forty‐three phospho‐site‐specific antibodies were spotted onto the antibody array. Measurement of signal detection was conducted in line with instructions from the manufacturer. Signal intensities of spots on the array were analysed via ImageJ (NIH, USA).

### 2.15 Surface plasmon resonance‐based binding analysis

The affinity determination of geniposide binding to TLR4 was measured by Biacore X100‐based surface plasmon resonance (SPR) technology with the BIA evaluation system (GE Healthcare, Sweden). SPR assay is performed by an extremely sensitive biosensor that offers rapid screening of the alterations of refractive index on a molecular‐immobilized chip in response to another small molecule in a real‐time and label‐free manner. It is a powerful quantitative tool to monitor biomolecular interactions and provide specific kinetic and affinity determination, including the dissociation constant (*K*_D_). In the Biacore X100 system, recombinant human TLR4 (R&D Systems, USA) is covalently immobilized on the surface of a CM5 chip by a coupling buffer (10‐mM sodium acetate, pH = 5) from the Amine Coupling Kit (GE Healthcare, Sweden). The coupling level was 1,300 RU. Then, a running buffer (PBS buffer mixed with 0.05% Triton X‐100, pH = 7.4) was prepared prior to the dilution of geniposide. Afterwards, TLR4‐immobilized chip was treated with a running buffer containing the geniposide with different concentrations (0–12.8 μM) followed by the regeneration scouting by 10‐mM glycine–HCl (pH = 1.7). The injection and dissociation time was set to 3 and 15 min, respectively. The binding affinity of geniposide to TLR4 was analysed by BIAevaluation (GE Healthcare, Sweden).

### 2.16 Data and statistical analysis

The data and statistical analysis comply with the recommendations of the *British Journal of Pharmacology* on experimental design and analysis in pharmacology. All the studies were designed to generate groups of equal sample size, using randomization and blinded analysis. Statistical analysis was undertaken only for studies where the sample size of each group was at least *n* = 5. The declared group size was the number of independent values, and the statistical analysis was done using these independent values by SPSS 21.0 (SPSS Inc., USA). The normality and homogeneity of variances were measured prior to statistical tests. For the comparison between two groups, Student's *t* test was used for normally distributed data, whereas Mann–Whitney *U* test was applied for data with non‐normal distribution. However, for the multiple comparisons (groups more than two), both the non‐parametric test (Kruskal–Wallis test) and one‐way ANOVA with Tukey's multiple comparisons test were used. Post hoc tests were run only if *F* achieved *P* < 0.05 in ANOVA and there was no significant variance inhomogeneity. Due to the reduction of unwanted variation, we used ‘fold‐matched control values’ to normalize the data of Western blotting and RT‐PCR. All the data are presented as mean ± SD. When the *P* value was less than 0.05, we considered it as evidence of a significant difference for all tests. *P* value for statistical significance was not varied later in Section 3. Meanwhile, all the outliers in our study were included in the data analysis and presentation.

### 2.17 Nomenclature of targets and ligands

Key protein targets and ligands in this article are hyperlinked to corresponding entries in http://www.guidetopharmacology.org, the common portal for data from the IUPHAR/BPS Guide to PHARMACOLOGY (Southan et al., 2016; Harding et al., 2018), and are permanently archived in the Concise Guide to PHARMACOLOGY 2019/20 Alexander, Fabbro, et al., 2019a, b; Alexander, Kelly, et al., 2019).

## 3 RESULTS

### 3.1 Geniposide suppresses in vivo primary and metastatic tumour growth in a mouse model of orthotopically implanted HCC

**FIGURE 1:**
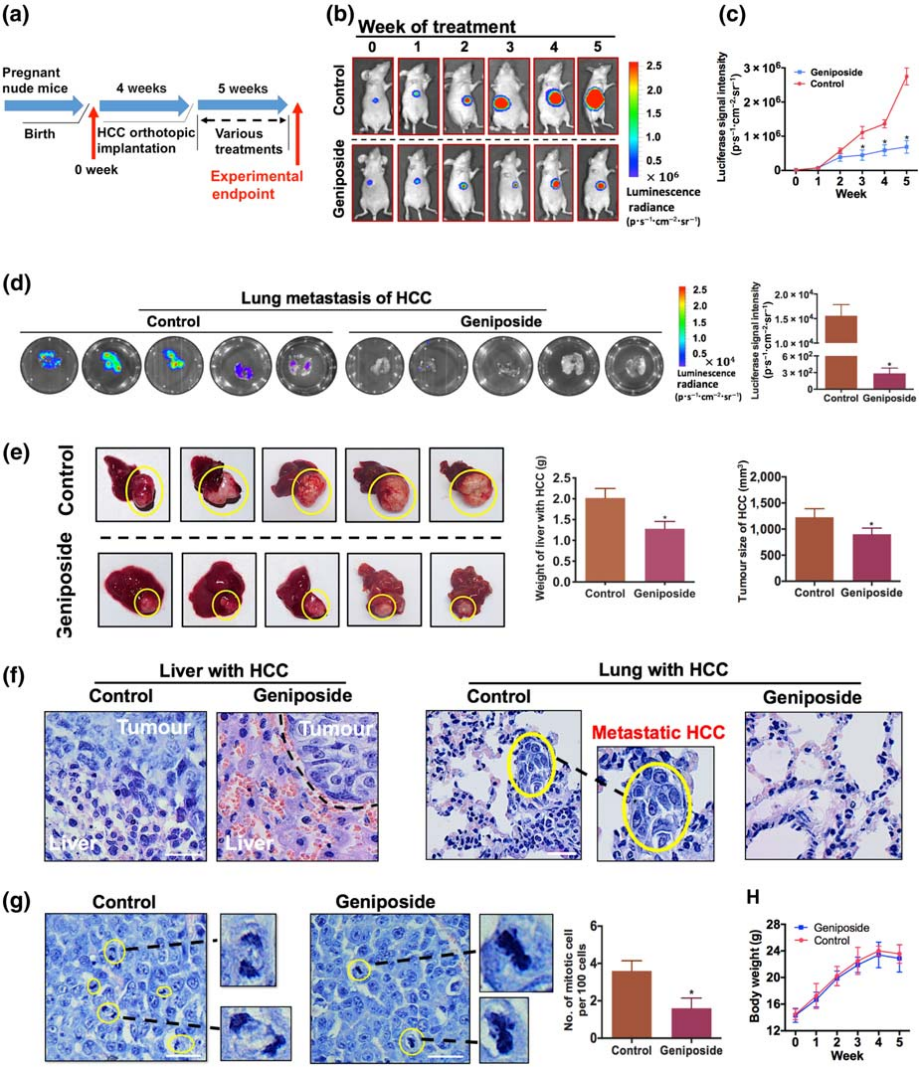
Geniposide attenuates primary and metastatic growth of hepatocellular carcinoma (HCC) in an orthotopic mouse model. (a) Schematic flowchart of the experimental procedure. (b) Representative figures of orthotopic implantation of luciferase‐tagged MHCC‐97L in mice with or without geniposide treatment (30 mg·kg^−1^ for 2 days). HCC growth was monitored, once a week, by capturing images of luciferase‐dependent bioluminescence in the hepatic region. (c) Detection of luciferase signal intensity in HCC mice. (d) Measurement of lung metastasis of luciferase‐expressing HCC and their comparison in signal intensity at the end of a 5‐week intervention. (e) Representative images of HCC‐bearing livers collected after growth for 5 weeks (left panel). Comparative results of the weight of livers with HCC and tumour size are respectively shown in the middle and right panels. (f) Haematoxylin and eosin staining of hepatic sections with invaded orthotopic HCC cells (left panel). The invasive edge between tumour and adjacent normal tissues has been illustrated by a black dotted line. Metastatic HCC cells were identified in the pulmonary tissue with yellow ellipses (right panel). The enlarged image nearby is the metastasized HCC in the lung. (g) Mitotic cells in orthotopic HCC are highlighted with a yellow ellipse and magnified in the lateral photograph. Quantification of mitotic events that emerged in HCC cells is presented in the bar chart (right panel). (h) Bodyweight of mice with or without geniposide treatment for 5 weeks. Note that geniposide treatment significantly attenuated HCC growth and pulmonary metastasis without cytotoxicity. All data are presented as mean ± SD of five independent assays with at least three replicates. ^*^ *P* < 0.05, significantly different from the control group. Scale bar, 20 μm for all images

We, first of all, identified the anti‐HCC potential of geniposide using an orthotopic HCC implantation model (Figure 1a). The model was established by implanting small cubes of subcutaneous‐grown HCC onto the right lobe of the liver in nude mice, as indicated in our previous study (Tan et al., 2015). Bioluminescence emitted from luciferase‐tagged MHCC‐97L represented the tumour size, which was weekly monitored, once a week for 5 weeks, in mice with vehicle (saline buffer) or oral geniposide treatment (30 mg·kg^−1^ for 2 days). Notably, progressively decreased signal intensity was observed in mice treated with geniposide in comparison with the control group from Week 3 onwards, suggesting the slower growth of orthotopic HCC under geniposide treatment (Figure 1b,c). More strikingly, after a 5‐week geniposide treatment, the severe distal lung metastasis of HCC was markedly repressed. The luciferase signal in the lung represented the metastasized HCC cells, which was stronger in the control group than in geniposide‐treated mice (Figure 1d). In addition, the endpoint detection of tumour size and weight of HCC‐bearing liver were measured, suggesting that HCC growth in the geniposide‐treated mice was significantly slower than that in the control group, in terms of tumour weight and volume (Figure 1e; *n* = 5 per group).

Consistent with the observation in bioluminescence images, the invasion, metastasis and proliferation of HCC were greater in mice without geniposide treatment. Firstly, orthotopic invasion of HCC exhibited an irregular and invasive edge in the growth front in the control mice, indicating that numerous HCC cells invaded into normal hepatic tissue. In contrast, a clear boundary was displayed in the mice on geniposide therapy (Figure 1f). Secondly, the absence of intrahepatic metastasis of HCC to the lung was clearly noted in the geniposide‐treated mice (Figure 1f). Thirdly, geniposide induced a marked attenuation of mitosis events that interspersed among HCC cells, which further supported the anti‐proliferation effect of geniposide (Figure 1g). Moreover, the reduction of body weight in the geniposide‐treated group was not significant, compared with the control group, indicating the non‐cytotoxic effects of geniposide (30 mg·kg^−1^ for 2 days) in treating animals (Figure 1h). Collectively, our findings showed that geniposide suppressed in vivo proliferation and lung metastasis of HCC, without apparent adverse effects (*n* = 5 per group).

### 3.2 Geniposide inhibited tumour angiogenesis without affecting HCC cell survival

**FIGURE 2:**
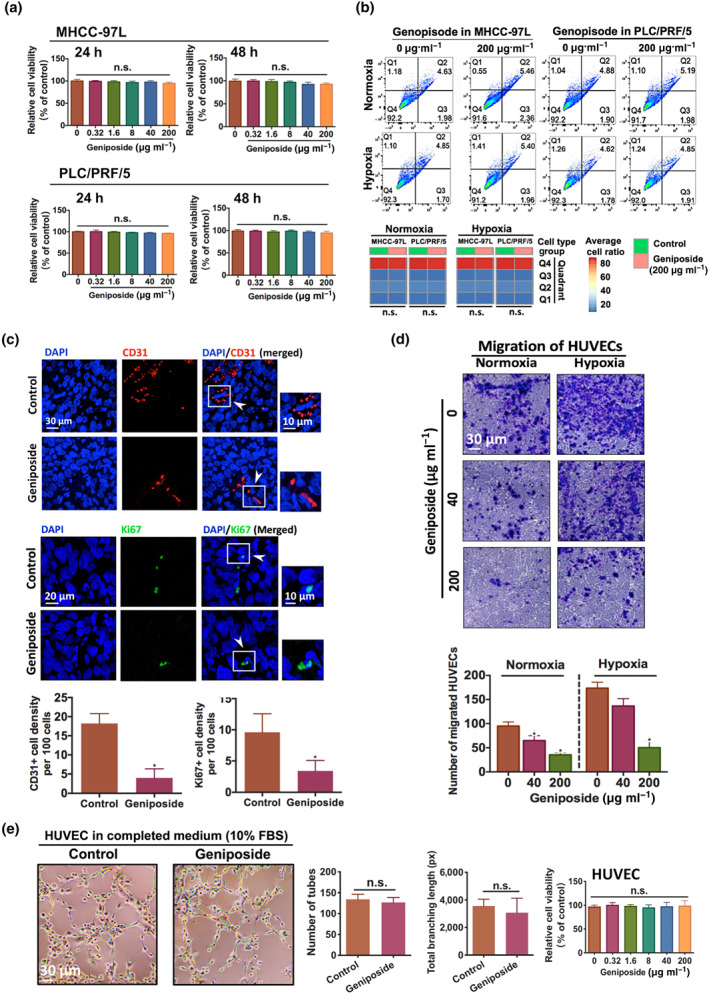
Geniposide suppressed tumour angiogenesis without inhibiting hepatocellular carcinoma (HCC) cell proliferation and survival. (a) Viability of MHCC‐97L and PLC/PRF/5 cells after geniposide treatment with concentration gradients (0.32–200 μg·ml^−1^) for 24 and 48 h under both normoxic and hypoxic conditions. (b) Detection of 200 μg·ml^−1^ of geniposide‐triggered apoptosis in MHCC‐97L and PLC/PRF/5 cells under normoxic and hypoxic conditions, by flow cytometry using double staining of PE Annexin V and 7‐ADD. Geniposide exerts non‐apoptotic effect on HCC cells. (c) Representative figures of HCC tissues from mice with or without geniposide administration (30 mg·kg^−1^ per 2 days). Antibodies against CD31 (endothelial cell marker) and Ki67 (proliferation marker) are utilized for immunostaining with Alexa Fluor 568 (red) and Alexa Fluor 488 (green), respectively. Cell nuclei are labelled with DAPI (blue). Blockade of CD31 and Ki67 expression can be apparently observed in mice with geniposide therapy. (d) Migration of HUVECs is significantly reduced when triggered by the supernatant derived from geniposide‐treated PLC/PRF/5 cells, under normoxic or hypoxic conditions, for 24 h. Based on transwell migration assay, the co‐culture system is established by culturing HUVECs in the donor chamber while geniposide‐stimulated PLC/PRF/5 cells are in the receiving chamber. (e) Measurement of tube formation of HUVECs with or without geniposide treatment (200 μg·ml^−1^) in non‐conditioned complete medium (EGM‐2 with FBS 10%) under normoxic condition. Quantification of tubular networks is shown in the bar charts (middle panel), while in the right panel, viability of geniposide‐treated HUVECs (0.32–200 μg·ml^−1^) for 24 h under normoxic condition is determined by MTT assay. Note that geniposide did not directly suppressing endothelial cell‐dependent angiogenesis when the culture condition is in the absence of HCC. All data are shown as mean ± SD of five independent assays with at least three replicates. ^*^ *P* < 0.05 significantly different from the control group; ^n.s.^No significant difference from control

Geniposide with concentration up to 200 μg·ml^−1^ exhibited no cytotoxic and apoptotic effects on both MHCC‐97L and PLC/PRF/5 cells under normoxic or hypoxic condition for 24‐ or 48‐h incubation (Figure 2a,b), which indicates that the in vivo inhibition of HCC growth and metastasis by geniposide was not associated with its cytotoxicity to HCC cells.

As tumour angiogenesis was associated with the progression and metastasis of HCC (G. B. Wang et al., 2010), we examined whether the in vivo repression of HCC tumour by geniposide was associated with angiogenesis inhibition. As shown in Figure 2c, the average intensity of positive CD31 (endothelial cell marker) or Ki67 (proliferation marker) signalling in hepatic tumour tissue was measured (J. Li et al., 2020). Spare CD31‐ or Ki67‐positive signals were detected in geniposide‐treated mice compared with that in control. Additionally, we established a co‐culture of HUVECs and PLC/PRF/5 cells in the transwell system by seeding HUVECs in the donor chamber while geniposide‐stimulated PLC/PRF/5 cells in the receiving chamber. Treatment of 24‐h co‐incubation under normoxic or hypoxic condition revealed that migration of HUVECs could be markedly suppressed in both conditions (Figure 2d; *n* = 5 per group). Geniposide, up to 200 μg·ml^−1^, showed neither cytotoxic nor any proliferative effects on HUVECs, as analysed by MTT assay (Figure 2e). Furthermore, the HUVEC tube formation assay showed that geniposide did not directly inhibit angiogenesis in the absence of HCC cells, cultured in the complete medium (10% FBS; Figure 2e). These results demonstrated that geniposide suppressed HCC angiogenesis by inhibiting the migration of endothelial cells in a non‐toxic manner.

### 3.3 Geniposide suppressed VEGF transcription by a HIF‐1α‐independent mechanism

**FIGURE 3:**
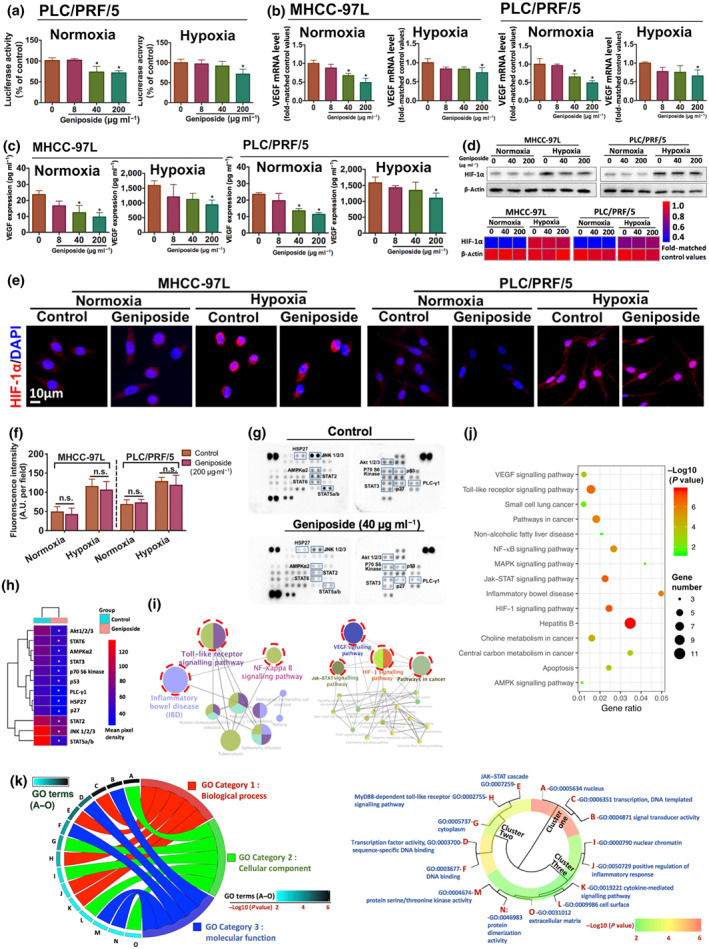
Geniposide inhibited VEGF transcription via HIF‐1α‐independent mechanisms. (a) Luciferase activities of geniposide‐treated PLC/PRF/5 cells under normoxic or hypoxic condition for 24 h are analysed by dual‐luciferase reporter assay system. PLC/PRF/5 cells are transfected with luciferase‐based pGL4.1 plasmid for the VEGF promoter region. Relative expression is indicated as percentages of control. (b) The mRNA profile of VEGF in MHCC‐97L and PLC/PRF/5 cells upon geniposide administration under oxygen‐containing or oxygen‐free conditions is assessed by RT‐PCR. Quantification of mRNA levels is obtained by the normalization to β‐actin. (c) The VEGF secretion levels from both MHCC‐97L and PLC/PRF/5 cells in response to geniposide stimulation under normoxic or hypoxic environments are measured by elisa assay. (d–f) Protein expression and nuclear translocation of HIF‐1α in MHCC‐97L and PLC/PRF/5 cells with or without geniposide treatment under normoxic or hypoxic condition are respectively measured by immunoblotting and immunofluorescence. (g) Whole‐cell lysates from PLC/PRF/5 cells with or without geniposide incubation for 24 h were collected for human phospho‐kinase array analysis. Relative expression of 43 phosphorylated‐specific antibodies spotted onto membranes was measured. The signal intensity with the statistical difference is highlighted with squares. (h) The expression of differential proteins validated in the phospho‐proteomic analysis is presented in the hierarchically clustered heatmap. (i) The network profile shows the prominently regulated pathways by geniposide enriched by CluoGO‐dependent clustering algorithm. Functional annotations of pathways are retrieved from KEGG database. (j) Scatterplot of the top 15 enriched KEGG pathways. The gene ratio represents the proportion of identified differential genes to the total number of genes involved in a specific pathway. (k) Circos plot in the left panel illustrates the associations between the top 5 enriched GO terms and three sub‐ontologies (BP, CC and MF) from the GO database. The correlation degree is represented as −log10 *P* value. In the right panel, the annotations of GO terms (A–O) are distributed into three principal embranchments in the cluster tree ranging from strong (red portion) to weak correlation (green portion). Note that geniposide significantly attenuates VEGF transcription by HIF‐1α‐independent mechanism. The anti‐VEGF action of geniposide is potentially related to the regulation of TLR4 and STAT3 signalling pathways in hepatocellular carcinoma cells. All data are indicated as mean ± SD of five independent experiments with at least three replicates. ^*^ *P* < 0.05 significantly different from the control group; ^n.s.^No significant difference versus control

Regarding the outcomes of the luciferase reporter assay, geniposide repressed the transcriptional activity of the VEGF promoter, after 24‐h treatment in the normoxic or in the hypoxic condition, in PLC/PRF/5 cells (Figure 3a). Furthermore, the transcriptional activity and secretion of VEGF in both MHCC‐97L and PLC/PRF/5 cells were dose‐dependently down‐regulated upon geniposide treatment under normoxic or hypoxic condition (Figure 3b,c), indicating that geniposide may potentially halt VEGF transcription activity without modulating hypoxia‐specific targets. As the expression of VEGF in hypoxia is mainly regulated by the activation of HIF‐1α (a transcription factor of VEGF), we further measured HIF‐1α expression in response to treatment with geniposide, in either normoxic or hypoxic conditions. The levels of HIF‐1α in HCC cells were not affected by geniposide treatment, suggesting that the decrease of VEGF induced by geniposide was not mediated by changes in HIF‐1α (Figure 3d–f).

### 3.4 Geniposide attenuates Sp1 and STAT3‐associated VEGF transcription in HCC cells

**FIGURE 4:**
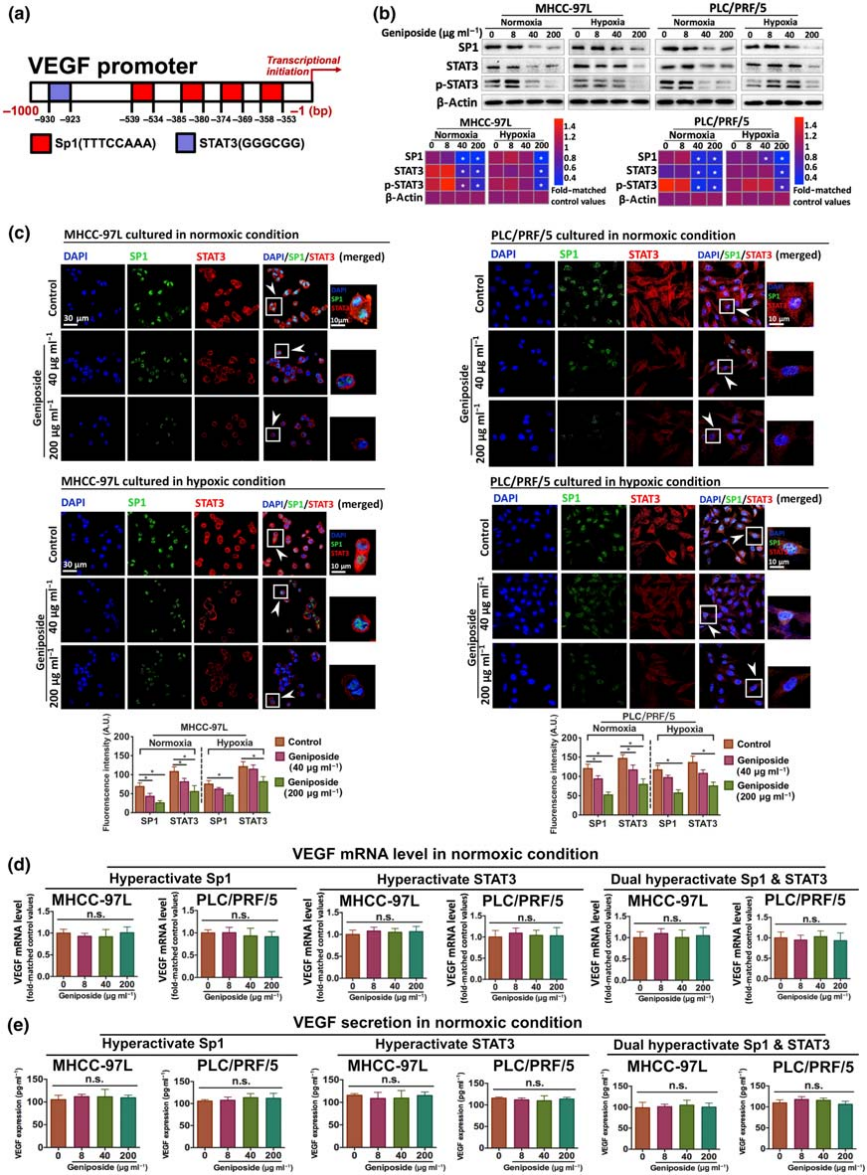
Geniposide down‐regulated Sp1‐ and STAT3‐related VEGF transcription in hepatocellular carcinoma (HCC) cells. (a) Binding sites of putative transcription factors (Sp1 and STAT3) in the specific sequence of luciferase‐targeted VEGF promotor. (b) Determination of expressions of Sp1, total and phosphorylated STAT3 in MHCC‐97L and PLC/PRF/5 cells with or without geniposide stimulation under normoxic or hypoxic condition. (c) Representative confocal images of in vitro HCC cells with or without geniposide. HCC slices are incubated with both antibodies, Sp1 and STAT3. Merged figures illustrate the co‐localization of Sp1 (green), STAT3 (red) and cell nucleus (blue). (d) Normoxic mRNA level or (e) secretory expression of VEGF in MHCC‐97L and PLC/PRF/5 cells with three independent conditions by plasmid transfection as follows: overexpression of Sp1, STAT3 and dual hyperactivation of Sp1 and STAT3, respectively. Note that the expression of Sp1 and STAT3 can be markedly inhibited by geniposide intervention. All data are shown as mean ± SD of five independent assays with at least three replicates. ^*^ *P* < 0.05 significantly different from the control group; ^n.s.^No significant difference versus control

A human phospho‐kinase array was used to analyse the phosphorylation profiles of 43 cancer‐associated kinases. Overexpressed spots on the antibody array are the potential targets, contributing to the final outcome (Figure 3g). Twelve significantly changed kinases in HCC cells under geniposide treatment (40 μg·ml^−1^) were hierarchically clustered in the heatmap (Figure 3h). To gain further details of the geniposide‐regulated molecules involved in the suppression of HCC, integrative bioinformatics analysis was applied for target validation, as described in our recent study (Tan et al., 2020), including gene‐annotation enrichment analysis from the KEGG and GO databases, GlueGO‐based network pharmacology and SPSS‐dependent cluster analysis. The top 15 KEGG pathways regulated by geniposide are shown in the scatter plot (Figure 3j), indicating that a large proportion of pathways were focused on mediating TLR/STAT/MAPK/NF‐κB‐related targets. Furthermore, the KEGG‐dependent ClueGO network was established in order to discount the irrelevant biological terms and to identify the strongly correlated pathways by kappa statistics (Figure 3i). The network revealed that regulation of the crosstalk between TLR/NF‐κB‐related inflammation (left panel) and STAT/HIF‐1/VEGF‐dependent tumour angiogenesis (right panel) might be the crucial pathways in geniposide‐induced suppression of HCC. However, as we had found that HIF‐1α stability was not influenced by geniposide treatment, the HIF‐1 pathway was excluded as a target in the subsequent bioinformatics analysis. Based on the GO analysis and R programming visualization, the top 5 GO terms in each GO ontology were respectively displayed in both circos plot and cluster tree (Figure 3k). For the SPSS‐dependent cluster tree, the GO terms, including GO Term A (−log10(*q*) = 4.783), GO Term B (−log10(*q*) = 4.625) and GO Term C (−log10(*q*) = 4.596), were statistically classified into Cluster 1 with high correlations (in red). More specifically, GO Term A (nucleus) was a single branch of Cluster 1. Besides, the GO Term B (signal transducer activity) and GO Term C (transcription) were conjugated together in another branch of Cluster 1, suggesting that the regulation of STAT (GO Term B + C) and nuclear target (GO Term A) may be responsible for the suppression of HCC by geniposide. Taken together, the inhibitory effect of geniposide on HCC angiogenesis was predictively mediated by STAT/NF‐κB/TLR‐related pathways with a potential nuclear target (Figure 3k). On the basis of the bioinformatics findings and literature review, Sp1 might be the candidate nuclear target. As an essential transcription factor located in the nucleus and known to regulate cell survival and angiogenesis, Sp1 is frequently overactivated during tumourigenesis (Liu, Du, Hu, Zhao, & Xia, 2018). The crosstalk between two transcription factors, Sp1 and STAT3, is essential for tumour angiogenesis and metastasis (Huang & Xie, 2012). Surprisingly, the transcription binding sites of Sp1 and STAT are involved in the luciferase‐tagged region of the VEGF promoter (−1 to −1,000 bp; Figure 4a; Pages & Pouyssegur, 2005). Therefore, geniposide‐induced inactivation of theVEGF promoter may result from the disruption of the binding potential of Sp1 and STAT3 to its transcriptional regions in the VEGF promoter.

In order to demonstrate, experimentally, the target predictions from the bioinformatics study, both immunoblotting and immunofluorescence assays were performed. Suppression of protein levels of Sp1, STAT3 and p‐STAT3 were observed in geniposide‐treated MHCC‐97L and PLC/PRF/5 cells under normoxic or hypoxic conditions (Figure 4b). Also, the immunofluorescence signalling intensities of Sp1 and STAT3 in both MHCC‐97L and PLC/PRF/5 cells had a downward trend after geniposide treatment (Figure 4c). Furthermore, plasmid transfection (pN3‐Sp1FL and EF.STAT3C.Ubc.GFP)‐induced overexpression of Sp1 or STAT3, or dual hyperactivation of Sp1 and STAT3, consistently resulted in neutralizing the anti‐VEGF effect of geniposide at mRNA and secretion level (Figure 4d,e). Based on these findings, we postulate that the inhibition of Sp1/STAT3‐associated VEGF transcription was the mechanism underlying geniposide‐induced suppression of HCC.

### 3.5 Antagonization of the TLR4/MyD88 pathway by TLR4–geniposide interaction suppresses VEGF expression in HCC cells

**FIGURE 5:**
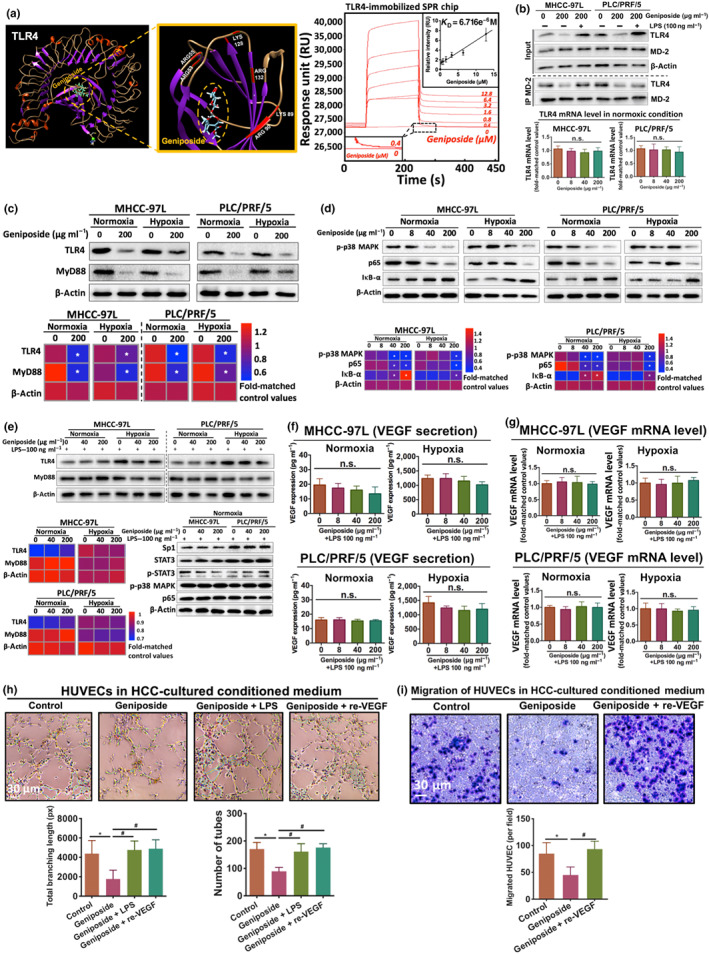
Direct inhibition of the TLR4/MyD88 pathway is involved in the anti‐angiogenic actions of geniposide in hepatocellular carcinoma (HCC). (a) 3D structure of binding profile between TLR4 and geniposide is visualized and analysed by in silico molecular docking approach. Magnified views are the potential binding clusters (left panel). The central figure is the sensorgram (red curves) for the binding affinity analysis of geniposide with various concentrations (0–12.8 μM) over a TLR4‐immobilized CM5 sensor chip, which is analysed by surface plasmon resonance (SPR) technology, while the right inset plot (black line) represents the response intensity of 0–12.8‐μM geniposide to TLR4. The linear regression is plotted by a four‐parameter logistic equation (*R* ^2^ = 0.952, *K*_D_ = 6.716e^−6^M) by the BIAevaluation system (GE Healthcare, Sweden). (b) The interaction of MD2 and TLR4 was measured by co‐immunoprecipitation assay and immunoblotting (upper panel), while the lower panel is the mRNA expression of TLR4 in both MHCC‐97L and PLC/PRF/5 cells under geniposide treatment in normoxic condition. (c, d) Protein expressions of TLR4/MyD88 and its downstream factors p‐p38 MAPK, p65 and IκB‐α in both 24‐h geniposide‐treated MHCC‐97L and PLC/PRF/5 under either normoxic or hypoxic condition. (e) Protein levels of TLR4, MyD88, Sp1, STAT3, p‐STAT3, p‐p38 MAPK and p65 in MHCC‐97L and PLC/PRF/5 cells under co‐treatment of geniposide and LPS (100 ng·ml^−1^). (f, g) Secretion and mRNA activity of VEGF in both MHCC‐97L and PLC/PRF/5 cells upon the cooperative intervention of geniposide and LPS (100 ng·ml^−1^) under normoxic or hypoxic environment, analysed by elisa assay and RT‐PCR, respectively. (h) Determination of HCC‐derived tube formation of HUVECs via either single or co‐treatments as shown in the left panel, including geniposide (200 μg·ml^−1^), LPS (100 ng·ml^−1^) and recombinant VEGF (re‐VEGF; 20 ng·ml^−1^). Quantification of tubular networks is shown in the bar charts (lower panel). (i) The migration of HUVECs is significantly reduced when cultured in the supernatant derived from geniposide‐treated PLC/PRF/5 cells under normoxic conditions for 24 h. Note that the TLR4/MyD88 signalling pathway can be down‐regulated by geniposide treatment, which is caused by geniposide‐induced direct inhibition of TLR4 protein. Also, the addition of either LPS or recombinant VEGF leads to significant reversal of the anti‐angiogenic effect of geniposide. All data are presented as mean ± SD of five independent experiments with at least three replicates. ^*^ *P* < 0.05 significantly different from the control group; ^#^ *P* < 0.05 significantly different from the geniposide group; ^n.s.^No significant difference versus control

Our bioinformatics study and published reports demonstrated that the TLR4/MyD88 signalling pathway plays a vital role in tumour suppression (Kang, Su, Sun, & Zhang, 2018; Murad, 2014). We, first of all, examined the interaction between geniposide and its predicted anti‐HCC targets by *in* silico molecular docking. Coincidentally, TLR4 exhibited the highest binding potency with geniposide (fill fitness: −3,318.93 kcal·mol^−1^; △*G*: −7.75 kcal·mol^−1^) by six residues, including *ARG53*, *ARG55*, *ARG90*, *ARG132*, *LYS89* and *LYS128* (Figure 5a). Furthermore, SPR technology was applied to determine experimentally the binding ability of geniposide with TLR4 (Figure 5a). The Biacore system‐based SPR analysis illustrated that geniposide could specifically bind to TLR4 (*K*_D_ = 6.716e^−6^M). The experimental data of steady‐state relative intensities (RU) fit well with a straight‐line regression model calculated by a four‐parameter logistic equation, with a positive correlation coefficient (*R* ^2^ = 0.952). Moreover, the mRNA expression of TLR4 in both MHCC‐97L and PLC/PRF/5 cells, with or without geniposide treatment (200 μg·ml^−1^) was measured by RT‐PCR (Figure 5b), indicating that no significant changes of the mRNA for TLR4 were observed in HCC cells. These results suggested that geniposide was not able to regulate the transcription of TLR4 and further consolidated the evidence for binding of geniposide to TLR4. Meanwhile, the exploratory interaction of MD2, also known as lymphocyte antigen 96, with TLR4 was determined by co‐immunoprecipitation assay (Figure 5b), which revealed that the TLR4–MD2 interaction was inhibited by geniposide‐induced TLR4 protein degradation but competitively antagonized by the addition of LPS (3 mg·kg^−1^ per single injection; H. Chen et al., 2019). The immunoblotting assay demonstrated that expression of TLR4 and its downstream molecules was inhibited by geniposide in HCC cells, including MyD88, p‐p38 MAPK, p65 and IκB‐α (Figure 5c,d). As the alteration of TLR4/MyD88 expression is commonly linked to the downstream regulation of STAT3 and Sp1 (J. X. Chen et al., 2017; Xiao et al., 2017), we examined if reactivation of TLR4 by its agonist LPS could reverse geniposide‐induced inhibition of STAT3 and Sp1 in HCC cells. Our experiments showed that LPS restored the expression of TLR4 and MyD88. Meanwhile, in the exploratory in vitro data, LPS further restored the downstream targets of TLR4/MyD88, including Sp1, STAT3, p38 MAPK and NF‐κB p65, in geniposide‐treated HCC cells (Figure 5e). Moreover, the geniposide‐induced attenuation of VEGF transcripts and secretion was neutralized by the addition of LPS (Figure 5f,g). In further experiments, the anti‐angiogenesis effect of geniposide in HCC was confirmed by endothelial cell tube formation assays (Figure 5h). The skeletonized tube‐like structures were abundant in cultures of HUVECs incubated in PLC/PRF/5 cell culture medium after geniposide treatment rather than that in geniposide–LPS or geniposide–re‐VEGF (20 ng·ml^−1^) co‐treatment (Figure 5h). Besides, the migration of HUVECs in PLC/PRF/5‐cultured medium was significantly reduced by geniposide administration, but its benefits were abolished when co‐treated with 20 ng·ml^−1^ of recombinant VEGF (Figure 5i). These findings suggested that geniposide suppressed the TLR4/MyD88 pathway leading to the inhibition of STAT3/Sp1‐dependent VEGF production in HCC cells.

### 3.6 In vivo reactivation of TLR4/MyD88 pathway attenuated the anti‐HCC effect of geniposide

**FIGURE 6:**
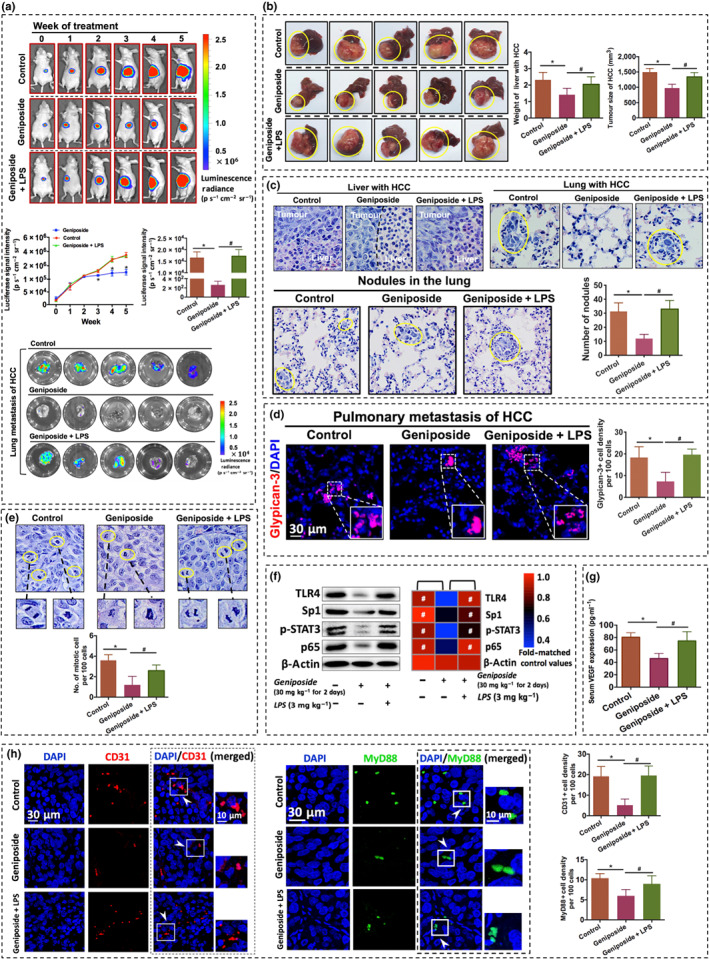
The in vivo suppression of orthotopic hepatocellular carcinoma (HCC) growth and lung metastasis via geniposide is mediated by the TLR4/MyD88 pathway. (a) Representative figures of orthotopic growth of MHCC‐97L cells expressing luciferase reporter (uppermost panel) from mice with various treatments, including vehicle, geniposide (30 mg·kg^−1^ for 2 days) and geniposide with LPS (3 mg·kg^−1^ in a single injection), respectively. Line chart and histogram in the middle panel illustrate the luciferase signal intensities of HCC in either primary (left) or metastatic regions in the lung (right), respectively. The representative image showing lung metastasis of luciferase‐labelled MHCC‐97L cells is in the lower panel. Tumour growth was monitored by luciferase imaging in live animals, once a week. (b) Representative images of HCC in the liver from control or LPS‐injected mice with geniposide treatment. Both the weights of HCC with liver tissue and HCC volume were measured as shown in the right panel, respectively. (c) Representative graphs show haematoxylin and eosin staining of orthotopic HCC developed in the livers and lungs from mice with different interventions. A boundary between tumour and normal liver is clearly noticeable in the group treated with geniposide alone. Quantification of pulmonary nodules is presented in the bar chart (lower panel). (d) Pulmonary metastasis of HCC identified by glypican‐3/DAPI co‐localization. Glypican‐3‐positive cells in the lungs of each group are quantified. (e) Mitotic events in HCC are presented in the left panel. Quantification of the mitotic index is shown in the right panel. (f) HCC‐derived in vivo protein expression of TLR4, Sp1, p‐STAT3 and p65 in the HCC mouse model with diversified treatments. (g) Serum VEGF expression in orthotopic HCC mice with three independent treatments, including vehicle, geniposide (30 mg·kg^−1^ for 2 days) and geniposide with LPS (3 mg·kg^−1^ per single injection), respectively. (h) Representative immunofluorescence graphs of hepatic tumour tissues from HCC‐bearing mice with various treatments. Inset images in the right corner are the respectively enlarged fields with merged signals, showing that either CD31 (red) or MyD88 (green) is overlapped with DAPI staining (blue). Note that targeting HCC angiogenesis by geniposide (30 mg·kg^−1^ for 2 days) is associated with the direct shutdown of the TLR4/MyD88‐dependent pathway, which was reversed by the additional administration of LPS (3 mg·kg^−1^ in a single injection). These results demonstrated that geniposide can significantly repress HCC proliferation, angiogenesis and pulmonary metastasis by down‐regulating the TLR4/MyD88 signalling pathway. All data indicated are means ± SD of five independent experiments with at least three replicates. ^*^ *P* < 0.05 significantly different from the control group; ^#^ *P* < 0.05 significantly different from the geniposide group. Scale bar, 30 μm for all images

To examine whether the inhibition of TLR4/MyD88 mediated the in vivo anti‐HCC effect of geniposide, orthotopic HCC mice were co‐treated with geniposide and LPS (3 mg·kg^−1^ per single injection; *n* = 5 per group). HCC signals were enhanced in mice co‐treated with LPS and geniposide, compared with those from the geniposide‐treated group on Week 5 (Figure 6a); There was no significant difference in luciferase signal intensity of primary and metastasized HCC in mice with vehicle or LPS–geniposide co‐treatment. Furthermore, endpoint observation illustrated that the tumour weight and size in HCC mice with LPS–geniposide co‐treatment were more substantial than that in geniposide‐alone‐treated mice, but there was no statistical difference when compared with control group (Figure 6b). Taken together, the addition of LPS blocked the inhibitory effect of geniposide on HCC proliferation and metastasis. Besides, as in the model group, the boundary between HCC and normal hepatic tissue was blurred in the mice co‐treated with geniposide and LPS (Figure 6c). Additionally, compared with geniposide‐treated mice, more lung metastases and mitotic events of HCC cells were noticed in mice with LPS–geniposide co‐treatment (Figure 6c–e). As shown in Figure 6d, lung metastasis of HCC was detected by an HCC‐specific biomarker, glypican‐3 (Zhou, Shang, Yu, & Tian, 2018). Moreover, in vivo protein expressions of targets in the TLR4/MyD88 pathway were restored in the mice co‐treated with geniposide and LPS, including TLR4, Sp1, p‐STAT3 and p65 (Figure 6f). Furthermore, LPS rescued the geniposide‐induced inhibition of serum VEGF expression in HCC mice (Figure 6g). The immunofluorescence assay demonstrated that CD31/MyD88‐positive cells in HCC sections were decreased in genipoiside‐treated mice but recovered after the addition of LPS (Figure 6h). These results suggested that reactivation of the TLR4/MyD88 pathway, by LPS could abolish the in vivo inhibitory effect of geniposide on HCC growth and angiogenesis.

## 4 DISCUSSION

Using the orthotopic mouse model, geniposide was shown to suppress growth of both primary and lung metastases of HCC. Interestingly, the in vitro experiments indicated that the anti‐HCC effects of geniposide in HCC mice were expressed without cytotoxicity. As VEGF is one of the critical factors that facilitate tumour angiogenesis, the inhibition of VEGF production, induced by geniposide, blocked the downstream effector signalling of VEGF and led to decrease in HCC growth. As an anti‐VEGF strategy, the VEGF monoclonal antibody is clinically effective in cancer treatment. Our study offers a potential and affordable anti‐VEGF, low MW, compound as an alternative approach to the treatment of HCC.

Although HIF‐1α‐triggered VEGF activation is a principal mechanism leading to angiogenesis in HCC, the inhibition of VEGF production in geniposide‐treated HCC was not accompanied by changes in HIF‐1α. This finding suggested that VEGF expression can be regulated independently of HIF‐1α. Notably, previous evidence indicated that, in the context of HCC growth, the Sp1‐related pathways can lead to the overexpression of VEGF, without altering HIF‐1α activity, because significant VEGF production has been shown in siHIF‐1α‐transfected Hep3B cells (an HCC cell line) under normoxic or hypoxic condition and neutralized after Sp1 silencing (Choi, Park, Song, & Choi, 2011). Both Sp1 and STAT3 are important transcription factors in the nucleus (Sp1) and cytoplasm (STAT3), respectively (Huang & Xie, 2012). In this study, we noted that geniposide‐induced inhibition of the Sp1/STAT3 pathway could suppress VEGF in HCC cells, HIF‐1α‐independently of HIF‐1α.. Re‐expression of Sp1 or STAT3, or both, significantly reactivated the expression of VEGF in geniposide‐treated HCC cells. Both factors can cooperatively promote VEGF‐dependent tumour angiogenesis (Santra, Santra, Zhang, & Chopp, 2008). As a zinc finger transcription factor, Sp1 is an inducer of tumour angiogenesis by directly binding to the VEGF promoter (Wu et al., 2013). Moreover, the transcriptional activity of the VEGF promoter in glioblastoma cells can be synergistically activated by STAT3 and Sp1 (Loeffler, Fayard, Weis, & Weissenberger, 2005). Simultaneous inhibition of Sp1 and STAT3 was reported to arrest the invasive and metastatic ability of HCC by down‐regulating VEGF expression (Zou et al., 2015). The data from our study confirmed the critical role of Sp1/STAT3 in VEGF‐mediated angiogenesis in HCC.

**FIGURE 7:**
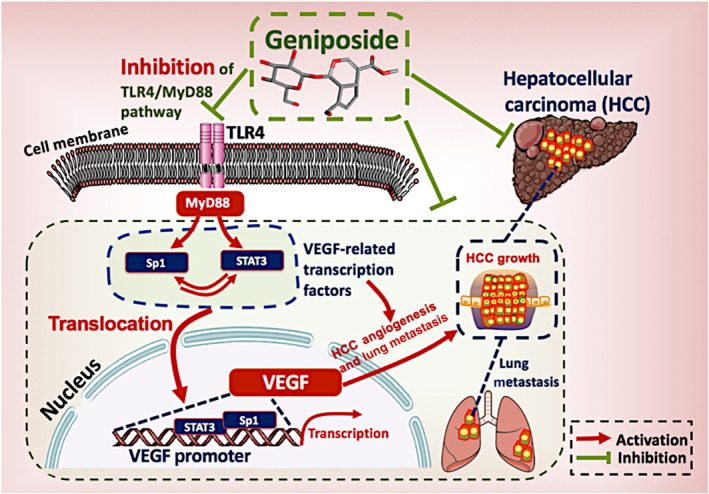
Diagram of the proposed mechanisms underlying the suppressive effects of geniposide on angiogenesis in HCC

TLR4, a member of the toll‐like receptor family, is involved in the responses of the innate and adaptive immune systems (Arias et al., 2018). However, TLR4 is not confined to immune cells but is found in numerous cancer cells and evokes pathological processes, including angiogenesis‐driven HCC promotion (Zhe et al., 2016). LPS can particularly bind to TLR4 and recruit the adaptor molecule MyD88 to initiate TLR4/MyD88 signal transduction, which consequently activates a series of downstream intracellular adaptors, kinases and transcription factors (Lu, Xu, Chen, Zhou, & Lin, 2017). As an agonist of TLR4, administration of LPS (3 mg·kg^−1^ per single injection) in HCC mice only aimed to reverse the geniposide‐induced inhibition of TLR4, instead of endotoxin‐triggered HCC progression in this study (Kitamura et al., 2002). Previous studies demonstrated that TLR4/MyD88 activation might up‐regulate STAT3, which accelerates proliferation, metastasis and multidrug resistance of HCC (Kang et al., 2018; F. Wang et al., 2019). Additionally, the activation of TLR4/MyD88 results in Sp1 accumulation in cancer progression (Dong et al., 2018). In our study, targeting TLR4/MyD88 could regulate both STAT3 and Sp1 in HCC growth, which could be blocked by geniposide treatment. in vitro and in vivo reactivation of the TLR4/MyD88 pathway by LPS in geniposide‐treated HCC did restore the tumour angiogenesis along with an increase of STAT3/Sp1‐dependent VEGF production, suggesting that modulation of STAT3/Sp1 in the TLR4/MyD88 pathway was critical to VEGF‐dependent HCC angiogenesis (Figure 7). Our study offers a novel mechanism to achieve VEGF inhibition in the treatment of HCC.

In conclusion, our study has demonstrated in vivo and in vitro activity, and the underlying mechanism, of geniposide against HCC. Geniposide exhibited potent inhibition of the proliferation, invasion, angiogenesis and lung metastasis of HCC. Suppression of HCC induced by geniposide was not associated with direct cytotoxicity in tumour cells but was related to a decrease of VEGF expression and HCC angiogenesis. Geniposide significantly blocked the transcription and production of VEGF from HCC cells. The inhibitory effect of geniposide on VEGF expression was independent of regulation by the HIF‐1α‐related pathway, but relied on suppression of Sp1 and STAT3 in HCC cells. Re‐expression of Sp1 and STAT3 could restore VEGF production in geniposide‐treated HCC cells. Further analysis showed that geniposide bound to TLR4 and repressed the TLR4/MyD88‐associated Sp1/STAT3 transcription activity. Re‐activation of the TLR4/MyD88 pathway by LPS, in vitro, restored the Sp1/STAT3 activities and VEGF production. Thus, geniposide may be a promising candidate for clinical use for HCC. Taken together, our study demonstrated that geniposide could be an inhibitor of angiogenesis in HCCby directly targeting the TLR4/MyD88‐regulated STAT3/Sp1 pathway followed by the suppression of VEGF transcription in a HIF‐1α independent manner, which provides a novel mechanism for VEGF inhibition in HCC management.

## AUTHOR CONTRIBUTIONS

Y.F. and N.W. conceived and designed the study. C.Z., N.W. and H.‐Y.T. did the experiments. C.Z., N.W., H.‐Y.T. and Y.F. wrote the manuscript. N.W., Z.Z., K.M., S.W.T., L. L. and Y.F. interpreted the data. All authors discussed and revised the manuscript and approved the final manuscript.

## CONFLICT OF INTEREST

The authors declare no conflicts of interest.

## DECLARATION OF TRANSPARENCY AND SCIENTIFIC RIGOUR

This Declaration acknowledges that this paper adheres to the principles for transparent reporting and scientific rigour of preclinical research as stated in the *BJP* guidelines for Design & Analysis, Immunoblotting and Immunochemistry, and Animal Experimentation, and as recommended by funding agencies, publishers and other organizations engaged with supporting research.
